# Fatigue in der Allgemeinbevölkerung: Ergebnisse der Studie „Gesundheit in Deutschland aktuell“ (GEDA 2023)

**DOI:** 10.1007/s00103-024-03950-1

**Published:** 2024-09-26

**Authors:** Christina Poethko-Müller, Angelika Schaffrath Rosario, Giselle Sarganas, Ana Ordonez Cruickshank, Christa Scheidt-Nave, Robert Schlack

**Affiliations:** 1 Abt. Epidemiologie und Gesundheitsmonitoring, FG Körperliche Gesundheit, Robert Koch-Institut, General-Pape-Str. 62–66, 12101 Berlin, Deutschland; 2 Abt. Epidemiologie und Gesundheitsmonitoring, FG Gesundheitsberichterstattung, Robert Koch-Institut, Berlin, Germany; 3 Abt. Epidemiologie und Gesundheitsmonitoring, FG Psychische Gesundheit, Robert Koch-Institut, Berlin, Germany

**Keywords:** Fatigue, Survey, Allgemeinbevölkerung, Soziodemografische Determinanten, Gesundheitsbezogene Determinanten, Fatigue, Survey, General population, Sociodemographic determinants, Health-related determinants

## Abstract

**Hintergrund:**

Fatigue ist ein Symptomkomplex, geht mit Müdigkeit, Energiemangel und Konzentrationsschwäche einher und hat durch Zusammenhänge mit Arbeitsunfähigkeit, Unfallgefährdung und erhöhten Bedarfen an Gesundheitsversorgung hohe Public-Health-Relevanz.

**Methode:**

Die Analysen basieren auf Daten von 9766 Erwachsenen des Surveys „Gesundheit in Deutschland aktuell (GEDA)“ 2023. Fatigue wurde mit der Fatigue Assessment Scale (FAS) erfasst, ein validiertes Instrument mit 10 Fragen zur Selbsteinschätzung von Fatigue. Die Skala wurde dichotomisiert in Ja (mindestens milde bis moderate Fatigue) versus Nein (keine Fatigue). Bevölkerungsgewichtete Prävalenzen von Fatigue und assoziierten soziodemografischen und gesundheitsbezogenen Faktoren wurden in deskriptiven Analysen und multivariabler Poisson-Regression berechnet.

**Ergebnisse:**

Die Prävalenz von Fatigue bei Erwachsenen in Deutschland beträgt 29,7 % (95 %-KI: 28,1–31,2), ist bei 18- bis 29-Jährigen am höchsten (39,6 % (95 %-KI: 35,0–44,4)) und nimmt in den Altersgruppen von 65 bis 79 Jahren ab (20,6 % (95 %-KI: 18,2–23,3)). In der Gruppe der Hochaltrigen liegt sie wieder höher (33,2 % (95 %-KI: 28,9–37,7)). Frauen haben ein höheres Risiko für Fatigue als Männer (adjustiertes relatives Risiko (aRR) 1,19 (95 %-KI: 1,08–1,32)). Fatigue ist unabhängig von Kovariablen signifikant mit Alter, niedrigerer Bildung, chronischer Erkrankung, Depressivität und Long Covid assoziiert.

**Diskussion:**

GEDA 2023 gehört zu den wenigen bevölkerungsbezogenen Studien, die Daten zur Fatigue erhoben haben. Die Ergebnisse ermöglichen Einschätzungen für Deutschland zur Häufigkeit von Fatigue und zur Bedeutung körperlicher, psychischer und sozialer Einflussfaktoren. Sie können als Referenz bzw. als Basis für zeitliche Trends im kontinuierlichen Gesundheitsmonitoring in Deutschland genutzt werden.

**Zusatzmaterial online:**

Zusätzliche Informationen sind in der Online-Version dieses Artikels (10.1007/s00103-024-03950-1) enthalten.

## Abstract (en)

**Background:**

Fatigue is an unspecific symptom complex characterized by tiredness, lack of energy, and lack of concentration and is of considerable public health relevance, due to its links with incapacity for work, risk of accidents, and increased need for healthcare.

**Methods:**

The analyses are based on data from 9766 adults of the telephone survey “Gesundheit in Deutschland aktuell (GEDA)” 2023. Fatigue was recorded using the Fatigue Assessment Scale (FAS), a validated instrument with 10 questions for self-assessment of fatigue. The scale was dichotomized into yes (at least mild to moderate fatigue) versus no (no fatigue). Population-weighted prevalences of fatigue and associated sociodemographic and health-related factors were calculated in descriptive analyses and multivariable Poisson regression.

**Results:**

The overall prevalence of fatigue in adults in Germany is 29.7% (95% CI 28.1–31.2), is highest in 18- to 29-year-olds (39.6% (95% CI 35.0–44.4)), and decreases in the age groups up to 65–79 years (20.6% (95% CI 18.2–23.3)). It is higher again in the very old age group (33.2% (95% CI 28.9–37.7)). Women have a higher risk of fatigue than men (aRR 1.19 (95% CI 1.08–1.32)). Fatigue is significantly associated with age, lower education, chronic illness, depression, and long COVID, regardless of covariates.

**Discussion:**

GEDA 2023 is one of the few population-based studies to have collected data on fatigue. The results allow estimates to be made for Germany on the frequency of fatigue and the significance of physical, psychological, and social influencing factors. They can be used as a reference or as a basis for trends over time as part of continuous health monitoring in Germany.

## Einleitung

### Hintergrund

Eine frühe, noch immer verwendete Definition beschreibt Fatigue als einen Zustand anhaltend empfundener Erschöpfung bei der Ausführung gewöhnlicher Tätigkeiten oder auch als ein Gefühl von Kraftlosigkeit und unzureichender Energie, um diese Tätigkeiten aufzunehmen [1]. Fatigue ist ein Symptomkomplex, der mit Müdigkeit, Schwäche, Energiemangel und Konzentrationsschwäche beschrieben wird bzw. einhergeht [2]. Anders als die Alltagsmüdigkeit kann Fatigue nicht durch übliche Strategien zur Wiederherstellung der Energie, wie beispielsweise Schlafen, gelindert werden und beeinträchtigt die Lebensqualität und Alltagsfunktionalität erheblich [3, 4]. Fatigue ist ein häufiger Beratungsanlass in der Primärversorgung und wird über den ICD-10-Code R53 (Unwohlsein und Ermüdung) erfasst. Im Gegensatz zur Diagnose „myalgische Enzephalopathie/chronisches Fatigue-Syndrom“ (ME/CFS; ICD-10-Code G93.3), für die explizite Diagnosekriterien vorliegen [5], handelt es sich bei Fatigue um einen unspezifischen Symptomenkomplex.

Fatigue tritt als Begleiterscheinung bei schweren Allgemeinerkrankungen auf, kann jedoch auch zahlreiche andere Ursachen haben. Die Diagnose und Behandlung von Fatigue sind daher mit großen Herausforderungen verbunden. Im Kontext epidemiologischer Studien ist Fatigue bislang wenig untersucht worden [6]. Sie wird nicht nur individuell als stark belastend empfunden [7], sondern ist darüber hinaus ein eigenständiger Prädiktor für Mortalität [8]. Unter gesellschaftlichen und ökonomischen Aspekten erlangt Fatigue vor allem durch die Zusammenhänge mit Einschränkungen der Lebensqualität [7], Arbeitsunfähigkeit [9], Unfallgefährdung [10] und den Bedarfen an Gesundheitsversorgung [3, 11] erhebliche Public-Health-Relevanz.

Seit der COVID-19-Pandemie hat Fatigue als eines der häufigsten über die akute SARS-CoV-2-Infektion hinaus fortbestehenden Symptome besondere Aufmerksamkeit erlangt [12]. Einschätzungen zur Prävalenz von Fatigue variieren dabei erheblich in Abhängigkeit von den Eigenschaften der Studienpopulation, der Länge der Nachbeobachtungsdauer seit der akuten Infektion und den zur Erfassung von Fatigue eingesetzten Erhebungsinstrumenten [13]. Studien zu den postakuten gesundheitlichen Folgen einer SARS-CoV-2-Infektion (Long Covid, Post-COVID-19-Zustand), die eine Kontrollgruppe nichtinfizierter Vergleichspersonen einschließen, bestätigen einen signifikanten Zusammenhang zwischen Fatigue und SARS-CoV-2-Infektion. Sie zeigen aber auch, dass nicht nur in der Gruppe der vormals SARS-CoV-2-Infizierten die Fatigue-Prävalenz hoch war, sondern auch in den Kontrollgruppen [14].

### Epidemiologie

Eine aktuelle systematische Übersichtsarbeit und Metaanalyse zur Prävalenz von Fatigue analysierte Daten aus 91 Studien, die weltweit bis 2021 durchgeführt wurden [6]. In die Analyse eingeschlossene Studien beziehen sich allerdings überwiegend nicht auf Zufallsstichproben der Allgemeinbevölkerung, sondern untersuchten Teilpopulationen beziehungsweise Subgruppen der Bevölkerung, insbesondere bestimmte Berufsgruppen. Insgesamt kamen bei diesen Studien 36 verschiedene Erhebungsinstrumente, darunter sehr unterschiedliche Fragebogeninstrumente, zur Erfassung von Fatigue zum Einsatz. Die Art des gewählten Erhebungsinstrumentes, aber auch die Erhebungsmethode (z. B. über schriftliche Fragebögen, Telefonbefragung oder persönliches Interview) hatten einen signifikanten Einfluss auf die Ergebnisse. Unteranalysen zeigten, dass Prävalenzschätzungen für Erwachsene in bestimmten Berufsgruppen mit 42,3 % (95 %-KI: 33,0–54,2) doppelt so hoch ausfielen wie für die erwachsene Allgemeinbevölkerung (20,4 % (95 %-KI: 16,7–25,0)). Zudem bestanden erhebliche Unterschiede nach Geschlecht, Studienregion und Zeitraum der Datenerhebung [6].

In Europa und speziell in Deutschland gibt es bislang nur wenige Studien, die bevölkerungsbasiert Aussagen zur Prävalenz von Fatigue treffen. Eine Studie mit aktueller Datengrundlage untersuchte eine Stichprobe von 2448 Erwachsenen im Alter von 45- bis 86 Jahren aus Lausanne (Schweiz) im Zeitraum von 2014 bis 2017 und schätzte die Prävalenz von Fatigue auf 21,9 % (95 %-KI 20,4–23,4). Zur Erfassung von Fatigue wurde die Fatigue Severity Scale (FSS) mit dem ursprünglich empfohlenen Schwellenwert von ≥ 4 Punkten verwendet [15]. 2 weitere europäische Studien in der erwachsenen Allgemeinbevölkerung wurden in Norwegen durchgeführt [16, 17]. Die ältere der beiden Studien [17] beruht auf im Jahr 1996 erhobenen Normdaten für 19- bis 80-Jährige für die Chalder-Fatigue-Scale, einem kurzen Fragebogen zur Erfassung der Schwere von Fatigue-Symptomen [18]. Nach den Ergebnissen dieser früheren Studie hatten insgesamt 11,4 % der Studienpopulation eine Fatigue, die mindestens über einen Zeitraum von mehr als 6 Monaten anhielt [17]. Die zweite norwegische Studie erhob im Jahr 2000 bei 19- bis 81-Jährigen eine Prävalenz von „high fatigue“ (FSS Score ≥ 5) von 23,1 % [16]. Mit dem initial empfohlenen Schwellenwert von ≥ 4 Punkten betrug die Prävalenz von Fatigue 46,7 %.

In Deutschland wurde die Prävalenz von Fatigue im Jahr 2004 mit der Chalder-Fatigue-Scale über persönliche Interviews bei 2591 Personen im Alter von 14–99 Jahren auf 14,2 % (95 %-KI: 12,8–15,6) eingeschätzt [19]. Für die Befragung war eine bundesweite bevölkerungsbezogene Stichprobe (*N* = 4165) gezogen worden. Ergebnisse einer 2020 publizierten Analyse zur zeitlichen Entwicklung in der Prävalenz von Müdigkeits- und Erschöpfungssymptomen bei Erwachsenen gründen sich auf den Einsatz der Kurzform des Gießener Beschwerdebogens in wiederholten bevölkerungsbezogenen Querschnittsuntersuchungen von Erwachsenen im Alter von 18–60 Jahren in den alten Bundesländern [16]. Die Ergebnisse zeigen, dass sich die geschätzte Prävalenz von Müdigkeit und von Erschöpfung über 3 Erhebungszeitpunkte (1975, 1994 und 2013) deutlich verändert hat. Die Prävalenzen waren für beide Symptome 1975 am höchsten (Müdigkeit 37,3 %; Erschöpfung 19,5 %), lagen 1994 bei 20,1 % bzw. 13,5 % und waren 2013 mit 21,9 % bzw. 15,3 % wieder etwas höher als 1994 [20].

Die Heterogenität bislang durchgeführter Studien zur Einschätzung der Prävalenz von Fatigue in der Bevölkerung ist groß. Die in Abhängigkeit von Studienpopulation, Operationalisierung von Fatigue und Erhebungszeitraum stark abweichenden Ergebnisse verdeutlichen den Bedarf an bevölkerungsrepräsentativen Studien, die eine verlässliche und über die Zeit vergleichbare Einschätzung zur Epidemiologie von Fatigue ermöglichen.

### Studienziele

Ziel der vorliegenden Studie war die Analyse bundesweiter, bevölkerungsbezogener Daten für eine aktuelle Bestandsaufnahme der Prävalenz von Fatigue und ihrer Determinanten bei Erwachsenen in Deutschland im Anschluss an die COVID-19-Pandemie.

## Methode

### Datenbasis: „Gesundheit in Deutschland aktuell“ (GEDA 2023)

Die Analysen umfassen Daten von 9766 Erwachsenen mit vollständigen Daten zur Fatigue Assessment Scale (FAS), dies entspricht 97,5 % von insgesamt 10.026 Teilnehmenden der Querschnittsstudie „Gesundheit in Deutschland aktuell“ (GEDA), die Informationen zum Gesundheitszustand und zu Einflussfaktoren der gesundheitlichen Lage von Personen ab 18 Jahren erhebt [21]. Die Datenerhebung erfolgte als standardisiertes computergestütztes telefonisches Interview in der Zeit vom 15.03.2023 bis 06.02.2024. Die Datenerhebung erfolgte durch Interviewende eines externen Markt- und Sozialforschungsinstituts (USUMA GmbH). Die Stichprobe wurde über das Dual-Frame-Verfahren gezogen, bei dem 2 Auswahlrahmen, Mobilfunk- und Festnetznummern, genutzt werden [22]. Die Responsequote wurde nach den Standards der American Association for Public Opinion Research (AAPOR) berechnet [23] und lag für einzelne Erhebungsmonate berechnet zwischen 17,4 % und 19,2 %.

### Zielgröße: Fatigue

Fatigue wurde mittels der FAS erhoben, die mit 10 Fragen die üblicherweise empfundene körperliche und psychische Müdigkeit erhebt. Die Bewertung erfolgt auf einer 5‑Punkte-Skala, die Antwortoptionen lauten niemals, manchmal, regelmäßig, oft, immer, mit einem Scoring von 1 (= nie) bis 5 (= immer). Die mögliche Gesamtpunktzahl der FAS reicht von 10 bis 50, wobei höhere Werte einen höheren Schweregrad der Fatigue anzeigen [24]. Die FAS wurde in den Niederlanden entwickelt und in der Allgemeinbevölkerung und bei Sarkoidose-Erkrankten validiert, aber auch bei anderen Krankheiten eingesetzt [25–27]. Als Schwellenwert für das Vorliegen einer mindestens milden bis moderaten Fatigue wird ein Summenscore von > 21 empfohlen. Von schwerer Fatigue wird bei einem FAS-Summenscore von > 34 ausgegangen. Der Grenzwert geht zurück auf Studien zur Untersuchung von Sarkoidose-bedingter Fatigue und wird national und international für Studien zu anderen Erkrankungen und bei Studien zu Long Covid angewendet [28–34].

### Determinanten

#### Soziodemografische Determinanten

Das Geschlecht wurde mit der Frage: „Welches Geschlecht wurde bei Ihrer Geburt in Ihre Geburtsurkunde eingetragen?“, erhoben. Das Lebensalter wurde kategorisiert in 18- bis 29 Jahre, 30- bis 44 Jahre, 45- bis 64 Jahre, 65- bis 79 Jahre, > 79 Jahre. Der höchste Bildungsabschluss des Haushaltes wurde anhand der CASMIN-Klassifikation in „einfach“, „mittel“ und „höher“ gruppiert [35]. Leben in Partnerschaft wurde mit der Frage: „Haben Sie einen festen Partner/eine feste Partnerin?“, erhoben.

#### Gesundheitsbezogene Determinanten

Der allgemeine Gesundheitszustand wurde mittels der Frage: „Wie ist Ihr Gesundheitszustand im Allgemeinen?“, in den Kategorien „sehr gut“, „gut“, „mittelmäßig“, „schlecht“ und „sehr schlecht“ abgefragt. Chronische Erkrankungen in den letzten 6 Monaten wurden über die Frage: „Haben Sie eine chronische Krankheit oder ein lang andauerndes gesundheitliches Problem? Damit gemeint sind Krankheiten oder gesundheitliche Probleme, die mindestens 6 Monate andauern oder voraussichtlich andauern werden“, erhoben. Das Screening auf depressive Symptomatik erfolgte über den Patient Health Questionnaire‑2 (PHQ-2): „Wie oft fühlten Sie sich im Verlauf der letzten 2 Wochen durch die folgenden Beschwerden beeinträchtigt: a) Niedergeschlagenheit, Schwermut oder Hoffnungslosigkeit? b) Wenig Interesse oder Freude an Ihren Tätigkeiten?“ Antwortmöglichkeiten waren jeweils „überhaupt nicht (0)“/„an einzelnen Tagen (1)“/„an mehr als der Hälfte der Tage (2)“/„beinahe jeden Tag (3)“. Ein positives Screening-Ergebnis liegt ab einem Score von 3 vor. Aktuell bestehendes Long Covid wurde wie folgt erfragt: „Haben Sie den Eindruck, dass bei Ihnen ein Long COVID oder Post-COVID-Zustand besteht oder bestanden hat?“ und „Besteht das Long COVID oder der Post-COVID-Zustand noch heute?“, und galt bei Bejahung der letztgenannten Frage als vorliegend.

### Statistische Auswertungen

Berechnet wurden Prävalenzen insgesamt sowie stratifiziert nach soziodemografischen und gesundheitsbezogenen Determinanten. Relative Häufigkeiten werden mit 95 %-Logit-Konfidenzintervallen (95 %-KI) angegeben, Gruppenunterschiede mit einem Chi-Quadrat-Test geprüft. Als statistisch signifikant gelten *p*-Werte kleiner als 0,01.

Für Zusammenhangsanalysen wurden relative Risiken (RR) über Poisson-Regressionen ohne sowie mit gegenseitiger Kontrolle der soziodemografischen und gesundheitsbezogenen Determinanten berechnet. In das gemeinsame Modell wurden nur Fälle einbezogen, für die vollständige Angaben zu allen Determinanten vorliegen.

Alle Auswertungen erfolgten insgesamt sowie stratifiziert nach Geschlecht. Dabei wurden Gewichtungsfaktoren verwendet, die sowohl die Auswahlwahrscheinlichkeiten der teilnehmenden Personen (auf Basis der Telefonnummern) als auch eine Anpassung an die Bevölkerung bezüglich Region, Alter, Geschlecht (Stand 31.12.2020) und Bildung (Mikrozensus 2018) berücksichtigen [22]. Absolute Anzahlen werden ungewichtet angegeben. Die Analysen wurden mit den Survey-Prozeduren von SAS 9.4 (SAS Institute Inc., Cary, NC, USA) und Stata 16.1 (StataCorp, 2019, College Station, TX, USA) durchgeführt.

## Ergebnisse

In die vorliegende Auswertung wurden 9766 Teilnehmende im Alter von 18 bis 107 Jahren (ungewichtetes Mittel: 59 Jahre, Median 61, gewichtetes Mittel 51, Median 52) eingeschlossen, davon waren 5219 (50,8 %) weiblich und 4547 (49,2 %) männlich (Tab. 1).

**Tab. 1:** Stichprobenbeschreibung gesamt und stratifiziert nach Geschlecht (ungewichtete Anzahl und ungewichtete und gewichtete Prozente)

		Gesamt			Frauen			Männer		
		Anzahl (ungewichtet)	Prozent (ungewichtet)	Prozent (gewichtet)	Anzahl (ungewichtet)	Prozent (ungewichtet)	Prozent (gewichtet)	Anzahl (ungewichtet)	Prozent (ungewichtet)	Prozent (gewichtet)
Geschlecht*	–	9766	–	100,0	5219	53,4	50,8	4547	46,6	49,2
Alter in Jahren	Anzahl	9766	–	–	5219	–	–	4547	–	–
	18–29 J.	833	8,5	16,3	341	6,5	15,5	492	10,8	17,1
	30–44 J.	1458	14,9	23,0	731	14,0	22,1	727	16,0	23,9
	45–64 J.	3603	36,9	34,9	1987	38,1	34,4	1616	35,5	35,4
	65–79 J.	2683	27,5	18,0	1494	28,6	18,8	1189	26,2	17,1
	80+	1189	12,8	7,9	666	12,8	9,3	523	11,5	6,5
Feste/r Partner/in	Anzahl	9740	–	–	5203	–	–	4537	–	–
	Ja	6510	66,8	61,3	3229	62,1	60,7	3281	72,3	62,0
	Nein	3230	33,2	38,7	1974	37,9	39,3	1256	27,7	38,0
	Missing	26	–	–	16	–	–	10	–	–
Subjektive Gesundheit	Anzahl	9756	–	–	5213	–	–	4543	–	–
	Sehr gut	2012	20,6	21,9	1017	19,5	21,6	995	21,9	22,2
	Gut	4770	48,9	46,0	2513	48,2	45,3	2257	49,7	46,7
	Mittelmäßig	2279	23,4	24,2	1296	24,9	25,3	983	21,6	23,0
	Schlecht	554	5,7	6,0	303	5,8	5,8	251	5,5	6,2
	Sehr schlecht	141	1,5	2,0	84	1,6	2,0	57	1,3	2,0
	Missing	10	–	–	6	–	–	4	–	–
Chronische Erkrankung	Anzahl	9746	–	–	5208	–	–	4538	–	–
	Ja	5197	53,3	51,2	2935	56,4	53,0	2262	49,9	49,3
	Nein	4549	46,7	48,8	2273	43,6	47,0	2276	50,2	50,7
	Missing	20	–	–	11	–	–	9	–	–
Depressive Symptomatik (PHQ 2)	Anzahl	9662	–	–	5153	–	–	4509	–	–
	Keine (PHQ-2: 0–2)	8347	86,4	82,7	4437	86,1	82,6	3910	86,7	82,9
	Depressive Symptomatik (PHQ-2: 3–6)	1315	13,6	17,3	716	13,9	17,4	599	13,3	17,1
	Missing	104	–	–	66	–	–	38	–	–
SARS-CoV-2-Infektion und Long-Covid‑/Post-Covid-Zustand (Selbstangabe)	Anzahl	9565	–	–	5097	–	–	4468	–	–
	Keine SARS-CoV-2-Infektion	3489	36,4	35,9	1857	36,4	33,1	1632	36,5	38,7
	SARS-CoV-2-Infektion, aktuell KEIN Long Covid	5492	57,4	57,4	2884	56,6	59,0	2608	58,4	55,8
	SARS-CoV-2-Infektion, aktuell Long Covid	584	6,1	6,7	356	7,0	8,0	228	5,1	5,4
	Missing	201	–	–	122	–	–	79	–	–

*Für Geschlecht sind Zeilenprozente angegeben, ansonsten Spaltenprozente

### Deskriptive Analysen

**Abb. 1:**
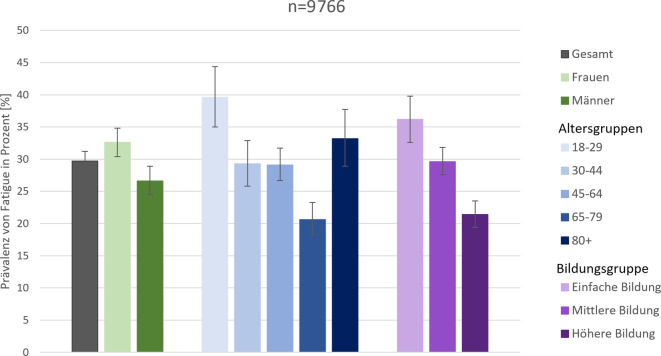
Prävalenz von Fatigue bei Erwachsenen stratifiziert nach Geschlecht, Altersgruppen und Bildungsgruppen nach der CASMIN-Klassifikation [35], Prävalenz (in %) und 95 %-Konfidenzintervalle, Quelle: GEDA 2023, eigene Abbildung

Die Prävalenz von mindestens milder bis moderater Fatigue beträgt nach den Daten von GEDA 2023 bei Erwachsenen in Deutschland 29,7 % (95 %-KI: 28,1–31,2) und ist mit 32,6 % bei Frauen höher als bei Männern mit 26,6 % (Abb. 1 und Tab. 2). Bei 18- bis 29-Jährigen ist die Prävalenz von Fatigue mit 39,6 % (95 %-KI: 35,0–44,4) am höchsten, in der Gruppe der 65- bis 79-Jährigen mit 20,6 % (95 %-KI: 18,2–23,3) am niedrigsten. In der Altersgruppe der Personen ab 80 Jahren beträgt sie 33,2 % (95 %-KI: 28,9–37,7). Ab 65 Jahren liegt die Prävalenz bei Frauen und Männern ähnlich hoch (Tab. 2). Das Muster im Altersverlauf unterscheidet sich kaum zwischen Frauen und Männern.

**Tab. 2:** Gewichtete Prävalenz Fatigue bei Erwachsenen gesamt und stratifiziert nach Geschlecht (gewichtete Prävalenzen, Determinanten, 95 %-Konfidenzintervalle, *p*-Werte), Quelle: GEDA 2023

		Gesamt					Frauen					Männer				
		Anzahl	Anzahl mit Fatigue	Prävalenz Fatigue (%)	(95 %-KI)	*p*-Wert	Anzahl	Anzahl mit Fatigue	Prävalenz Fatigue (%)	(95 %-KI)	*p*-Wert	Anzahl	Anzahl mit Fatigue	Prävalenz Fatigue (%)	(95 %-KI)	*p*-Wert
Geschlecht	Gesamt	9766	2314	29,7	(28,1–31,2)	0,0002	5219	1364	32,6	(30,4–34,8)	–	4547	950	26,6	(24,5–28,9)	–
	Männer	4547	950	26,6	(24,5–28,9)	–	–	–	–	–	–	–	–	–	–	–
	Frauen	5219	1364	32,6	(30,4–34,8)	–	–	–	–	–	–	–	–	–	–	–
Alter in Jahren	Gesamt	9766	–	–	–	< 0,0001	5219	–	–	–	< 0,0001	4547	–	–	–	0,0001
	18–29 J.	833	288	39,6	(35,0–44,4)	–	341	139	44,0	(37,1–51,2)	–	492	149	35,5	(29,5–41,9)	–
	30–44 J.	1458	362	29,3	(25,8–32,9)	–	731	216	32,9	(28,2–38,1)	–	727	146	25,7	(21,0–31,1)	–
	45–64 J.	3603	869	29,1	(26,7–31,7)	–	1987	552	32,9	(29,5–36,5)	–	1616	317	25,3	(22,0–29,0)	–
	65–79 J.	2683	455	20,6	(18,2–23,3)	–	1494	261	21,7	(18,3–25,5)	–	1189	194	19,4	(16,0–23,5)	–
	80+	1189	340	33,2	(28,9–37,7)	–	666	196	33,4	(27,8–39,4)	–	523	144	32,9	(26,5–39,9)	–
Bildung (CASMIN)	Gesamt	9738	–	–	–	< 0,0001	5205	–	–	–	< 0,0001	4533	–	–	–	< 0, 0001
	Einfache Bildung	1453	458	36,2	(32,6–39,8)	–	755	251	40,2	(35,2–45,5)	–	698	207	32,7	(28,0–37,7)	–
	Mittlere Bildung	4236	1109	29,6	(27,6–31,8)	–	2486	682	31,1	(28,3–34,0)	–	1750	427	27,9	(24,8–31,2)	–
	Höhere Bildung	4049	742	21,4	(19,4–23,5)	–	1964	426	27,0	(23,7–30,5)	–	2085	316	16,4	(14,2–18,8)	–
Feste/r Partner/in	Gesamt	9740	–	–	–	< 0,0001	5203	–	–	–	0,0006	4537	–	–	–	< 0,0001
	Ja	6510	1310	25,9	(24,1–27,8)	–	3229	747	29,6	(27,0–32,3)	–	3281	563	22,1	(19,7–24,8)	–
	Nein	3230	1003	35,8	(33,2–38,6)	–	1974	616	37,3	(33,8–41,0)	–	1256	387	34,2	(30,3–38,4)	–
Subjektive Gesundheit	Gesamt	9756	–	–	–	< 0,0001	5213	–	–	–	< 0,0001	4543	–	–	–	< 0,0001
	Sehr gut	2012	134	10,4	(8,2–13,1)	–	1017	70	12,1	(8,6–16,6)	–	995	64	8,7	(6,2–12,1)	–
	Gut	4770	760	21,3	(19,4–23,5)	–	2513	438	24,1	(21,3–27,2)	–	2257	322	18,5	(15,9–21,6)	–
	Mittelmäßig	2279	936	47,8	(44,5–51,1)	–	1296	575	51,1	(46,7–55,5)	–	983	361	44,0	(39,1–49,0)	–
	Schlecht	554	364	72,5	(66,8–77,5)	–	303	209	74,2	(66,9–80,4)	–	251	155	70,8	(61,9–78,3)	–
	Sehr schlecht	141	117	84,2	(72,5–91,5)	–	84	70	89,3	(79,0–94,9)	–	57	47	78,9	(57,8–91,1)	–
Chronische Erkrankung	Gesamt	9746	–	–	–	< 0,0001	5208	–	–	–	< 0,0001	4538	–	–	–	< 0,0001
	Ja	5197	1699	40,7	(38,4–42,9)	–	2935	1033	44,6	(41,5–47,6)	–	2262	666	36,3	(33,1–39,7)	–
	Nein	4549	610	18,1	(16,2–20,1)	–	2273	328	19,0	(16,4–21,9)	–	2276	282	17,2	(14,7–20,1)	–
Depressive Symptomatik (PHQ 2)	Gesamt	9662	–	–	–	< 0,0001	5153	–	–	–	< 0,0001	4509	–	–	–	< 0,0001
	Keine depressive Symptomatik (PHQ-2:0–2)	8347	1407	21,1	(19,6–22,7)	–	4437	845	23,9	(21,7–26,1)	–	3910	562	18,3	(16,3–20,5)	–
	Depressive Symptomatik (PHQ-2: 3–6)	1315	871	70,1	(66,2–73,8)	–	716	496	74,0	(68,7–78,7)	–	599	375	66,0	(60,1–71,5)	–
Long-Covid‑/Post-Covid-Zustand (Selbstangabe)	Gesamt	9565	–	–	–	< 0,0001	5097	–	–	–	< 0,0001	4468	–	–	–	< 0,0001
	Keine SARS-CoV-2-Infektion	3489	854	30,1	(27,6–32,8)	–	1857	468	30,7	(27,3–34,3)	–	1632	386	29,6	(26,0–33,5)	–
	SARS-CoV-2-Infektion, aktuell KEIN Long-Covid‑/Post-Covid-Zustand	5492	1076	25,2	(23,3–27,2)	–	2884	640	29,1	(26,4–32,0)	–	2608	436	21,0	(18,5–23,7)	–
	SARS-CoV-2-Infektion, aktuell Long-Covid‑/Post-Covid-Zustand	584	303	56,0	(49,3–62,4)	–	356	194	54,9	(46,2–63,2)	–	228	109	57,6	(47,3–67,4)	–

Anzahlen ungewichtet, Prozentangaben gewichtet

Bei beiden Geschlechtern ist die Prävalenz von Fatigue in der Gruppe mit einfacher Bildung am höchsten und am niedrigsten in der Gruppe mit höherer Bildung (Frauen 40,2 % vs. 27,0 %; Männer 32,7 % vs. 16,4 %; Tab. 2). Die Prävalenz von Fatigue ist bei Personen in fester Partnerschaft insgesamt um 10 Prozentpunkte niedriger als bei Personen ohne feste Partnerschaft (25,9 % vs. 35,8 %), der Unterschied ist bei Männern (22,1 % vs. 34,2 %) größer als bei Frauen (29,6 % vs. 37,3 %). Bei chronisch kranken Frauen und Männern ist die Prävalenz höher als bei Erwachsenen ohne chronische Erkrankung (40,7 % vs. 18,1 %). Fatigue ist mit 70,1 % (95 %-KI: 66,2–73,8) bei bestehender depressiver Symptomatik um mehr als das 3‑Fache häufiger als bei Erwachsenen ohne depressive Symptomatik (21,1 % (95 %-KI: 19,6–22,7)). Die Prävalenz von Fatigue bei Erwachsenen, die aktuell Long Covid angeben, wurde mit 56,0 % (95 %-KI: 49,3–62,4) doppelt so hoch geschätzt wie bei Erwachsenen nach SARS-CoV-2-Infektion ohne Long Covid (25,2 % (95 %-KI: 23,3–27,2)) bzw. bei Erwachsenen ohne SARS-CoV-2-Infektion (30,1 % (95 %-KI: 27,6–32,8)).

### Zusammenhangsanalysen

Frauen haben auch bei Kontrolle für die anderen soziodemografischen und gesundheitsbezogenen Determinanten ein um 19 % höheres Risiko für Fatigue als Männer (aRR 1,19 (95 %-KI: 1,08–1,32); Tab. 3). Wie in den deskriptiven Auswertungen zeigen sich auch in den adjustierten Analysen bei beiden Geschlechtern Zusammenhänge zwischen Fatigue und den soziodemografischen Determinanten Alter und Bildung. Bei Frauen wie bei Männern ist das Risiko für Fatigue deutlich mit jüngerem und mittlerem Lebensalter korreliert. Das höhere Risiko im jungen und mittleren Erwachsenenalter ist bei Frauen stärker ausgeprägt als bei Männern. Die Bedeutung von Bildung scheint sich zwischen den Geschlechtern zu unterscheiden: Während für Männer ein höheres Risiko für Fatigue bei einfacher Bildung (aRR 1,37 (95 %-KI: 1,11–1,68)) und mittlerer Bildung (aRR 1,28(95 %-KI 1,07–1,52)) im Vergleich zu höherer Bildung besteht, besteht dieses bei Frauen nur für die Gruppe mit einfacher Bildung (aRR 1,28 (95 %-KI 1,06–1,54)). Bei beiden Geschlechtern sind die Zusammenhänge zwischen Fatigue und chronischer Erkrankung, Depressivität und Long Covid auch in der adjustierten Analyse statistisch signifikant. Generell sind die Zusammenhänge in der adjustierten Analyse etwas weniger stark ausgeprägt als in der deskriptiven Analyse, mit Ausnahme des Zusammenhangs mit dem Alter.

**Tab. 3:** Gewichtete relative Risiken mit (aRR) und ohne Adjustierung (RR) gesamt und stratifiziert nach Geschlecht (RR/aRR, 95 %-Konfidenzintervalle, *p*-Werte), Quelle: GEDA 2023

		Gesamt				Frauen				Männer			
		Relatives Risiko, ohne Adjustierung (95 %-KI) *n* = 9766	*p*-Wert, ohne Adjustierung	Relatives Risiko, adjustiert (95 %-KI) *n* = 9401	*p*-Wert, adjustiert	Relatives Risiko, ohne Adjustierung (95 %-KI) *n* = 5219	*p*-Wert, ohne Adjustierung	Relatives Risiko, adjustiert (95 %-KI) *n* = 4997	*p*-Wert, adjustiert	Relatives Risiko, ohne Adjustierung (95 %-KI) *n* = 4547	*p*-Wert, ohne Adjustierung	Relatives Risiko, adjustiert (95 %-KI) *n* = 4404	*p*-Wert, adjustiert
Geschlecht	Gesamt	–	0,0002	–	0,0003	–	–	–	–	–	–	–	–
	Männer	Referenz		Referenz		–	–	–	–	–	–	–	–
	Frauen	1,22 (1,10–1,36)		1,19 (1,08–1,32)		–	–	–	–	–	–	–	–
Alter in Jahren	Gesamt	–	< 0,0001	–	< 0,0001	–	< 0,0001	–	< 0,0001	–	0,0001	–	< 0,0001
	18–29 J.	1,92 (1,61–2,28)		2,48 (2,09–2,94)		2,03 (1,61–2,55)		2,68 (2,15–3,35)		1,82 (1,40–2,37)		2,20 (1,68–2,89)	
	30–44 J.	1,42 (1,19–1,69)		1,83 (1,56–2,16)		1,52 (1,22–1,90)		1,97 (1,61–2,41)		1,32 (1,00–1,74)		1,66 (1,27–2,15)	
	45–64 J.	1,41 (1,21–1,64)		1,44 (1,25–1,65)		1,52 (1,25–1,85)		1,60 (1,35–1,90)		1,30 (1,03–1,65)		1,24 (0,98–1,55)	
	65–79 J.	Referenz		Referenz		Referenz		Referenz		Referenz		Referenz	
	80+	1,61 (1,34–1,93)		1,45 (1,23–1,70)		1,54 (1,21–1,96)		1,36 (1,11–1,67)		1,69 (1,28–2,24)		1,64 (1,28–2,11)	
Bildung (CASMIN)	Gesamt	–	< 0,0001	–	0,0012	–	0,0001	–	0,0181	–	< 0,0001	–	0,0060
	Einfache Bildung	1,69 (1,48–1,94)		1,29 (1,13–1,49)		1,49 (1,24–1,79)		1,28 (1,06–1,54)		2,00 (1,63–2,45)		1,37 (1,11–1,68)	
	Mittlere Bildung	1,39 (1,23–1,56)		1,13 (1,01–1,27)		1,15 (0,99–1,35)		1,04 (0,90–1,21)		1,70 (1,42–2,04)		1,28 (1,07–1,52)	
	Höhere Bildung	Referenz		Referenz		Referenz		Referenz		Referenz		Referenz	
Feste/r Partner/in	Gesamt	–	< 0,0001	–	0,0699	–	0,0006	–	0,1743	–	< 0,0001	–	0,1205
	Ja	Referenz		Referenz		Referenz		Referenz		Referenz		Referenz	
	Nein	1,39 (1,25–1,54)		1,10 (0,99–1,21)		1,26 (1,11–1,44)		1,09 (0,96–1,25)		1,55 (1,31–1,82)		1,14 (0,97–1,33)	
Chronische Erkrankung	Gesamt	–	< 0,0001	–	< 0,0001	–	< 0,0001	–	< 0,0001	–	< 0,0001	–	< 0,0001
	Ja	2,25 (1,99–2,54)		1,84 (1,63–2,09)		2,35 (2,00–2,76)		1,99 (1,69–2,35)		2,11 (1,76–2,53)		1,67 (1,39–2,02)	
	Nein	Referenz		Referenz		Referenz		Referenz		Referenz		Referenz	
Depressive Symptomatik (PHQ 2)	Gesamt	–	< 0,0001	–	< 0,0001	–	< 0,0001	–	< 0,0001	–	< 0,0001	–	< 0,0001
	Keine depressive Symptomatik (PHQ-2: 0–2)	Referenz		Referenz		Referenz		Referenz		Referenz		Referenz	
	Depressive Symptomatik (PHQ-2: 3–6)	3,32 (3,03–3,64)		2,67 (2,41–2,95)		3,10 (2,77–3,47)		2,46 (2,16–2,80)		3,61 (3,13–4,18)		2,88 (2,46–3,37)	
Long-Covid‑/Post-Covid-Zustand (Selbstangabe)	Gesamt	–	< 0,0001	–	< 0,0001	–	< 0,0001	–	0,0394	–	< 0,0001	–	< 0,0001
	Keine SARS-CoV-2-Infektion	Referenz		Referenz		Referenz		Referenz		Referenz		Referenz	
	SARS-CoV-2-Infektion, aktuell KEIN Long-Covid‑/Post-Covid-Zustand	0,84 (0,75–0,94)		0,93 (0,83–1,04)		0,95 (0,82–1,10)		0,99 (0,85–1,14)		0,71 (0,59–0,85)		0,87 (0,72–1,03)	
	SARS-CoV-2-Infektion, aktuell Long-Covid‑/Post-Covid-Zustand	1,86 (1,61–2,15)		1,36 (1,17–1,59)		1,79 (1,47–2,17)		1,25 (1,03–1,52)		1,95 (1,57–2,42)		1,63 (1,30–2,04)	

Anzahlen ungewichtet, relative Risiken gewichtet

## Diskussion

### Kernergebnisse.

In der vorliegenden bevölkerungsbezogenen Studie mit telefonischer Befragung einer Zufallsstichprobe von Erwachsenen in Deutschland wird die bevölkerungsgewichtete Prävalenz von Fatigue im Jahr 2023 auf insgesamt 29,7 % geschätzt. Die Prävalenz von Fatigue war bei Frauen höher als bei Männern, in jungem und mittlerem Erwachsenenalter höher als bei Erwachsenen im Alter zwischen 65 und 79 Jahren und bei Erwachsenen mit niedriger Bildung höher als bei Erwachsenen mit mittlerer oder hoher Bildung. Deskriptive Analysen und multivariable Regressionsanalysen zeigen einen Zusammenhang von Fatigue mit chronischer Erkrankung, depressiver Symptomatik und Long Covid. Auch unter Kontrolle dieser möglichen Störfaktoren bleiben die soziodemografischen Zusammenhänge mit dem Geschlecht, jüngerem Lebensalter und niedrigerem Bildungsniveau bestehen, wobei der Bildungseffekt in der adjustierten Analyse schwächer ausgeprägt ist.

### Einordnung in nationale und internationale Studien

#### Gesamtprävalenz.

Der direkte Vergleich mit Fatigue-Prävalenzen in anderen Ländern [15–17, 36] wird erschwert durch die methodische Heterogenität der Studien unter anderem in Bezug auf das Alter der Teilnehmenden, Erhebungszeit, die eingesetzten Instrumente und die gewählten Grenzwerte [37]. So bietet die in den Jahren zwischen 1994 und 2017 in Westeuropa berichtete Spanne von Fatigue in der Allgemeinbevölkerung von 11,4–38,0 % (46,7 % in Norwegen mit ursprünglichem Grenzwert des FSS) wenig Möglichkeit, die absolute Höhe der in GEDA 2023 erhobenen Prävalenz von 29,7 % zu bewerten.

#### Geschlecht und Alter.

Die höhere Prävalenz bei Frauen im Vergleich zu Männern, die sich aktuell in den GEDA-2023-Daten zeigt, bestätigt die Ergebnisse einer Vielzahl von sowohl älteren [16, 17, 19, 20, 36, 38] als auch neueren Studien [15, 39–41], die unabhängig von den eingesetzten Instrumenten eine höhere Betroffenheit von Frauen zeigen [6]. In unseren Analysen bestand der größte Geschlechtsunterschied in der Altersgruppe der 30- bis 64-Jährigen. In der schwedischen Monica-Studie zeigten sich die Unterschiede am ausgeprägtesten in der Gruppe der 45- bis 54-Jährigen [40]. In beiden Studien liegt der Gipfel der Geschlechtsdifferenz damit in der Lebensphase mit der höchsten Belastung durch Familien- und Berufsarbeit und zeigt möglicherweise einen Effekt der bekannten Ungleichverteilung von Wochenarbeitszeit unter Berücksichtigung der Familienarbeit („gender care gap“) an, mit deutlich höheren Anteilen unbezahlter Arbeit bei Frauen als bei Männern [42].

Weniger eindeutig ist die Studienlage zur Prävalenz von Fatigue im Altersverlauf. Während eine Reihe von Studien mit unseren Ergebnissen übereinstimmt, dass mit steigendem Alter die Prävalenz von Fatigue rückläufig ist und vor allem Erwachsene im jüngeren und mittleren Lebensalter von Fatigue betroffen sind [15, 39, 40], gibt es auch Studien, die entgegengesetzte Ergebnisse erbracht [17, 19, 38] oder keine Altersunterschiede festgestellt haben [1]. Anders als in dieser Studie, die eine monodimensionale Fatigue-Skala einsetzte, wurden in verschiedenen internationalen Studien mehrdimensionale Fatigue-Skalen (z. B. MF-20) mit differenzierbaren Dimensionen wie allgemeine, physische und mentale Fatigue eingesetzt. So zeigen sich in der Monica-Studie aus Schweden auf Basis von Daten aus 2014 signifikante altersabhängige Unterschiede mit höherer Betroffenheit im jüngeren Erwachsenenalter vor allem bei allgemeiner Fatigue und mentaler Fatigue [40], während sich bei der Skala zur körperlichen Fatigue zwar dieselbe Richtung der Zusammenhänge zeigte, der Zusammenhang jedoch sehr viel schwächer ausgeprägt war. Norwegische Daten aus den 1990er-Jahren ([17]; Chalder Scale) zeigten für Männer und Frauen unterschiedlich ausgeprägte Zusammenhänge von Fatigue mit dem Alter. Während für Männer ein deutlicher Anstieg der Prävalenz mit zunehmendem Alter zu beobachten war, zeigten sich bei Frauen in mittlerem Alter, ähnlich wie in unserer Studie, niedrigere Prävalenzen als bei jüngeren Frauen. Nur in der Altersgruppe der über 60- bis 80-Jährigen waren die Prävalenzen höher. Auch bei Männern gab es Zusammenhänge mit häufigerer Betroffenheit von Fatigue in höherem Alter nur hinsichtlich körperlicher Fatigue, während sich die Prävalenz mentaler Fatigue nicht zwischen den Altersgruppen unterschied [17]. Eine höhere Prävalenz von Fatigue im Alter zeigt sich in der vorliegenden Studie erst in der Gruppe der Über-79-Jährigen. Diese hochaltrige Altersgruppe wurde jedoch weder in der Monica-Studie noch in der o. g. norwegischen Studie von Loge et al. [17] untersucht. Möglicherweise zeigt der in GEDA 2023 erst für das sehr hohe Alter festgestellte Wiederanstieg der Prävalenz einen Effekt für das frühe und mittlere Rentenalter, der in der Schweizer Arbeit von Galland et al. postuliert wird und besagt, dass es einen säkularen Trend hin zu besserer Gesundheit im Rentenalter gibt [15], der Unterschiede zwischen älteren und neueren Studien erklären könnte.

Lerdal et al. [16] zeigten anhand von Daten aus Norwegen aus dem Jahr 2000, dass sich abhängig vom Vorliegen bzw. Nichtvorliegen einer chronischen somatischen Erkrankung die für Geschlecht und Bildung kontrollierten Mittelwerte für Fatigue (gemessen mit der FSS) stark unterschieden, innerhalb der Gruppe der chronisch kranken bzw. nicht chronisch kranken Personen jedoch keine Unterschiede nach Altersgruppen bestanden [16]. Unsere Analysen zeigen auch unter Kontrolle von Geschlecht, Bildung, chronischer Erkrankung und Depressivität signifikant niedrigere Prävalenzen für Fatigue mit zunehmendem Alter (mit Ausnahme der Hochaltrigen). Auch explorative, nach chronischer Erkrankung stratifizierte Sensitivitätsanalysen zeigen den Zusammenhang mit dem Alter in beiden Gruppen deutlich (Tab. 1S im Onlinematerial). Insgesamt scheint es ein komplexes Zusammenspiel zwischen somatischen Erkrankungen und dem Alter zu geben. Dabei zeigen sich jedoch in den verschiedenen Studien durchaus unterschiedliche Ergebnisse, die eine eindeutige Interpretation nicht zulassen.

#### Bildung und Partnerschaft.

Unabhängig von den eingesetzten Fatigue-Instrumenten und dem Alter der Studien zeigen sich in Studien aus verschiedenen Ländern deutliche Zusammenhänge zwischen Bildung und Fatigue, wobei Menschen mit niedriger formaler Bildung stärker von Fatigue betroffen waren [18, 19, 23, 33, 35, 36]. In einigen Studien, die zwischen mentaler und physischer Fatigue differenzieren, zeigte sich der Zusammenhang deutlicher bei der physischen Fatigue [17, 40]. Die letztgenannten Befunde können einen Zusammenhang zwischen vermehrter, mit schwerer körperlicher Arbeit verbundener Berufstätigkeit bei Personen mit niedriger Bildung nahelegen. Insgesamt zeigten unsere Analysen klar eine höhere Betroffenheit von Fatigue in Gruppen mit einfacher Bildung, insbesondere bei Männern. Fatigue kann damit ein Faktor sein, der gesundheitliche Ungleichheiten verstärkt, da infolge von Fatigue gesundheitsfördernde Aktivitäten und gesellschaftliche Teilhabe weniger stattfinden. Welche Faktoren für die Prävention von Fatigue hier im Detail eine Rolle spielen können, sollte Gegenstand zukünftiger Untersuchungen sein.

Das Leben in Partnerschaft zeigt sich in unseren Daten deskriptiv als eine Ressource, die mit geringerer Fatigue verbunden ist, und steht damit in Übereinstimmung mit einer Studie aus Dänemark [43]. Auch in einer US-amerikanischen Studie wurde gezeigt, dass verheiratete Personen seltener Fatigue berichteten als Unverheiratete [39], und es wurden geteilte Verantwortung und Arbeitsteilung von Alltagspflichten als mögliche Ursache diskutiert.

#### Gesundheitsbezogene Determinanten.

Die deskriptiven Analysen unserer Studie zeigen, wie auch die neueren Studienergebnisse aus Schweden und der Schweiz, dass ein als gut selbsteingeschätzter Gesundheitszustand deutlich mit geringerer Prävalenz von Fatigue verbunden ist [15, 40]. Auch die höhere Prävalenz bei Vorliegen von chronischen Erkrankungen steht im Einklang mit anderen europäischen Studien [16, 43]. Müdigkeit ist ein häufiges und für Betroffene sehr belastendes Symptom bei länger bestehenden Erkrankungen und muss nicht notwendigerweise durch den Krankheitsprozess selbst verursacht sein; erschöpfte psychosoziale Kompensationsmöglichkeiten, Schmerz, gestörter Schlaf, Folgen körperlicher Inaktivität und deren Wechselwirkungen untereinander werden als bedeutender diskutiert [44]. Sensitivitätsanalysen unserer Daten zeigen, dass das Fehlen einer festen Partnerschaft ausschließlich in der Gruppe chronisch Erkrankter mit einem höheren Risiko für Fatigue assoziiert ist (Tab. 1S im Onlinematerial).

Ein enger Zusammenhang zwischen psychischen Störungen, v. a. von Depression und Angststörungen, und Müdigkeit wurde in vielen Studien gezeigt [44, 45]. Müdigkeit ist ein Hauptsymptom der Depression und muss nicht mit Schlafstörungen assoziiert sein [46]. Verschiedene Hypothesen versuchen diesen Zusammenhang zu erklären: 1. Die Kriterien für chronische Müdigkeit und Depressionen überschneiden sich; 2. es besteht ein kausaler Zusammenhang, d. h., Menschen mit unerklärlicher chronischer Müdigkeit könnten primär an Depression leiden; 3. die Depression ist eine sekundäre Reaktion auf chronische Müdigkeit und Erschöpfung und 4. Confounding, d. h., gemeinsame Faktoren der Entstehung von Depression bzw. Müdigkeit sind nicht ausreichend berücksichtigt [47, 48]. Unsere Auswertungen zeigen die bekannten Zusammenhänge zwischen Fatigue und Depressivität.

#### Long Covid.

Fatigue ist eines der am häufigsten beschriebenen Symptome von Long Covid oder eines Post-Covid-Syndroms [49] und ist in vielen Studien, auch im Vergleich zu Kontrollgruppen ohne SARS-CoV-2-Infektion, untersucht worden. Angesichts der fast vollständigen Infektionsprävalenz von mindestens einer SARS-CoV-2-Infektion in der Bevölkerung, häufig auch unbemerkt, kann eine sichere Differenzierung zwischen Infizierten und nichtinfizierten Personen in bevölkerungsbezogenen Studien nur noch schwer vorgenommen werden. Die Bedeutung postinfektiöser Fatigue hat allerdings vor dem Hintergrund der großen Anzahl von SARS-CoV-2-Infektionen in der Bevölkerung innerhalb weniger Jahre deutlich zugenommen.

In GEDA 2023 wurde die subjektive Einschätzung erhoben, ob ein Long Covid vorliegt. Unter Bezugnahme auf diese Variable konnte untersucht werden, ob eine höhere Betroffenheit von Fatigue bei einem selbstberichteten Long-Covid-Zustand vorliegt. Wie in Studien mit anderen Long-Covid-Definitionen stellten wir eine deutlich höhere Fatigue-Prävalenz bei Personen mit selbstberichtetem Long Covid fest als bei Personen ohne diese Angabe. Bei der Interpretation dieses durchaus plausiblen Zusammenhanges muss jedoch bedacht werden, dass hieraus keine quantitativen Einschätzungen abgeleitet werden können. Analog zu Befragungssurveys im Vereinigten Königreich zur Prävalenz von Long Covid in der Bevölkerung [50] wurde in GEDA 2023 die subjektive Einschätzung zum Vorliegen von Long Covid oder Post-Covid-Zustand erfragt. Dies ist der Tatsache geschuldet, dass eine allgemein konsentierte Definition von Long Covid weiterhin nicht zur Verfügung steht. Es muss ausdrücklich betont werden, dass eine Post Exertional Malaise (PEM) als Kardinalsymptom für ein chronisches Fatigue-Syndrom (ME/CFS) mit dem FAS nicht erfasst wird. Rückschlüsse auf ein mögliches Vorliegen von ME/CFS, das symptomatologisch eine Überlappung mit einem Long- bzw. Post-Covid-Zustand aufweist [51], sind daher nicht möglich.

Auch wenn die Erkenntnisse zu Long- und Post-Covid aktuell noch immer zunehmen und einheitliche Definitionen oder allgemein konsentierte Diagnosekriterien derzeit noch fehlen, ist die Beobachtung der Entwicklung in den kommenden Jahren nicht nur wichtig, um eine möglicherweise zunehmende Krankheitslast durch Fatigue im Zusammenhang mit Long Covid zu erkennen, sondern auch um mittel- und langfristige gesundheitliche Entwicklungen der gesamten Bevölkerung nach mehr als 3 Jahren Pandemiebelastung mit in den Fokus des Gesundheitsmonitorings zu stellen.

### Stärken und Schwächen

GEDA 2023 gehört zu den wenigen aktuellen europäischen Studien, die die Häufigkeit von Fatigue in der Allgemeinbevölkerung beschreiben und gesundheitliche und soziodemografische Determinanten analysieren. Damit stehen für Deutschland aktuelle Daten zur Prävalenz der Fatigue über die gesamte Altersspanne des jungen Erwachsenenalters bis zur Hochaltrigkeit zur Verfügung. Die Studie hat Einschränkungen, die bei der Interpretation berücksichtigt werden müssen. So lässt das Querschnittsdesign keine Rückschlüsse auf mögliche Kausalitäten zu. Wie bei anderen telefonischen Befragungen ist die Response mit knapp unter 20 % gering und eine Beeinflussung der Ergebnisse durch die geringere Teilnahme von sehr kranken Menschen auf der einen Seite oder sehr gesunden, aktiven Menschen auf der anderen Seite kann nicht ausgeschlossen und in Richtung und Ausmaß nicht sicher abgeschätzt werden. Es ist davon auszugehen, dass einige Personengruppen unterrepräsentiert sind, wie z. B. Personen der niedrigen Bildungsgruppe, Menschen mit Migrationsgeschichte oder Menschen mit Beeinträchtigungen und Behinderungen. Einer selektiven Teilnahme wurde durch das Gewichtungsverfahren bestmöglich entgegengewirkt. Weiter gibt es bislang keine Festlegung oder Übereinkunft dazu, welche Instrumente Fatigue am besten erfassen. Die FAS hat keinen konkreten Zeitbezug, somit kann nicht differenziert werden, ob die Fatigue kürzer bzw. länger als 6 Monate besteht. Die Datenerhebung erfolgte telefonisch, obwohl die FAS ursprünglich als schriftlicher Fragebogen konzipiert wurde. Des Weiteren konnten weder Informationen zu Schichtarbeit oder Medikamentengebrauch berücksichtigt werden.

## Fazit

Die adjustierten Auswertungen unserer Studie zeigen, dass die Zusammenhänge von Geschlecht, Alter und niedrigerer Bildung mit Fatigue unabhängig von Depressivität, Long Covid und chronischen Erkrankungen bestehen bleiben. Sie können als Referenz bzw. als Basis für zeitliche Trends im Rahmen des kontinuierlichen Gesundheitsmonitorings in Deutschland genutzt werden. Die Zusammenhänge mit soziodemografischen Variablen und Parametern der körperlichen und mentalen Gesundheit geben wichtige Hinweise auf besonders von Fatigue betroffene Gruppen.

## Supplementary Information

Tabelle 1S: Gewichtete relative Risiken mit (aRR) und ohne Adjustierung (RR) gesamt und stratifiziert nach chronischer Erkrankung (RR/aRR, 95 %-Konfidenzintervalle, *p*-Werte), Quelle: GEDA 2
