# Supplementary material for: Fatigue in the general population: results of the “German Health Update 2023” study
Source: Bundesgesundheitsblatt Gesundheitsforschung Gesundheitsschutz. 2024 Sep 26;67(11):1208–21. [Article in German] doi: 10.1007/s00103-024-03950-1 (PMC11549105; doi:10.1007/s00103-024-03950-1)
Supplement: Supplementary file 1 — Tabelle 1S: Gewichtete relative Risiken mit (aRR) und ohne Adjustierung (RR) gesamt und stratifiziert nach chronischer Erkrankung (RR/aRR, 95 %-Konfidenzintervalle, p-Werte), Quelle: GEDA 2 [file 103_2024_3950_MOESM1_ESM.pdf]

Onlinematerial zum Beitrag
„Fatigue in der Allgemeinbevölkerung: Ergebnisse der Studie „Gesundheit in Deutschland aktuell“ (GEDA 2023)“
im Bundesgesundheitsblatt 2024

Christina Poethko-Müller, Angelika Schaffrath Rosario, Giselle Sarganas, Ana Ordonez Cruickshank, Christa Scheidt-Nave, Robert Schlack

Abteilung Epidemiologie und Gesundheitsmonitoring, Robert Koch-Institut, Berlin, Deutschland

Tabelle 1S Gewichtete relative Risiken mit (aRR) und ohne Adjustierung (RR) gesamt und stratifiziert nach chronischer Erkrankung (RR/aRR, 95 %-Konfidenzintervalle, p-Werte), Quelle: GEDA 2023

Tabelle 1S Gewichtete relative Risiken mit (aRR) und ohne Adjustierung (RR) gesamt und stratifiziert nach chronischer Erkrankung (RR/aRR, 95 %-Konfidenzintervalle, p-Werte), Quelle: GEDA 2023

		Gesamt				Mit chronischer Erkrankung				Ohne chronische Erkrankung			
		Relatives Risiko, ohne Adjustierung (95%-KI) n = 9.766	p-Wert, ohne Adjustierung	Relatives Risiko, adjustiert (95%-KI) n = 9.401	p-Wert, adjustiert	Relatives Risiko, ohne Adjustierung (95%-KI) n = 5.197	p-Wert, ohne Adjustierung	Relatives Risiko, adjustiert (95%-KI) n = 4.969	p-Wert, adjustiert	Relatives Risiko, ohne Adjustierung (95%-KI) n = 4.549	p-Wert, ohne Adjustierung	Relatives Risiko, adjustiert (95%-KI) n = 4.431	p-Wert, adjustiert
Geschlecht	Gesamt		0,0002		0,0003								
	Männer	Referenz		Referenz		Referenz		Referenz		Referenz		Referenz	
	Frauen	1,22 (1,10-1,36)		1,19 (1,08-1,32)		1,23 (1,10-1,37)	0,0004	1,22 (1,10-1,35)	0,0001	1,10 (0,89-1,37)	0,375	1,17 (0,94-1,45)	0,162
Alter in Jahren	Gesamt		<0,0001		<0,0001		<0,0001		<0,0001		<0,0001		<0,0001
	18-29 J.	1,92 (1,61-2,28)		2,48 (2,09-2,94)		2,04 (1,67-2,49)		1,95 (1,61-2,35)		3,68 (2,45-5,52)		3,71 (2,41-5,73)	
	30-44 J.	1,42 (1,19-1,69)		1,83 (1,56-2,16)		1,65 (1,37-1,99)		1,78 (1,50-2,12)		2,15 (1,41-3,29)		2,14 (1,36-3,35)	
	45-64 J.	1,41 (1,21-1,64)		1,44 (1,25-1,65)		1,55 (1,33-1,81)		1,46 (1,27-1,69)		1,41 (0,92-2,17)		1,48 (0,95-2,30)	
	65-79 J.	Referenz		Referenz		Referenz		Referenz		Referenz		Referenz	
	80+	1,61 (1,34-1,93)		1,45 (1,23-1,70)		1,63 (1,36-1,96)		1,44 (1,21-1,70)		1,59 (0,97-2,61)		1,52 (0,96-2,43)	
Bildung (CASMIN)	Gesamt		<0,0001		0,0012		<0,0001		0,0040		0,0536		0,228
	Einfache Bildung	1,69 (1,48-1,94)		1,29 (1,13-1,49)		1,59 (1,38-1,84)		1,28 (1,10-1,47)		1,29 (0,94-1,77)		1,30 (0,93-1,81)	
	Mittlere Bildung	1,39 (1,23-1,56)		1,13 (1,01-1,27)		1,37 (1,20-1,56)		1,14 (1,01-1,30)		1,32 (1,05-1,66)		1,11 (0,89-1,38)	
	Höhere Bildung	Referenz		Referenz		Referenz		Referenz		Referenz		Referenz	
Feste/r Partner/in	Gesamt		<0,0001		0,0699		<0,0001		0,0042		0,1357		0,836
	Ja	Referenz		Referenz		Referenz		Referenz		Referenz		Referenz	
	Nein	1,39 (1,25-1,54)		1,10 (0,99-1,21)		1,35 (1,21-1,50)		1,16 (1,05-1,29)		1,18 (0,95-1,47)		0,98 (0,77-1,23)	
Chronische Erkrankung	Gesamt		<0,0001		<0,0001								
	Ja	2,25 (1,99-2,54)		1,84 (1,63-2,09)									
	Nein	Referenz		Referenz									
Depressive Symptomatik (PHQ2)	Gesamt		<0,0001		<0,0001		<0,0001		<0,0001		<0,0001		<0,0001
	Keine depressive Symptomatik (PHQ-2: 0-2)	Referenz		Referenz		Referenz		Referenz		Referenz		Referenz	
	Depressive Symptomatik (PHQ-2: 3-6)	3,32 (3,03-3,64)		2,67 (2,41-2,95)		2,61 (2,37-2,89)		2,51 (2,26-2,79)		3,70 (3,00-4,57)		3,10 (2,42-3,97)	
Long COVID-/Post COVID-Zustand (Selbstangabe)	Gesamt		<0,0001		<0,0001		<0,0001		0,0002		<0,0001		<0,029
	Keine SARS-CoV-2-Infektion	Referenz		Referenz		Referenz		Referenz		Referenz		Referenz	
	SARS-CoV-2-Infektion, aktuell KEIN Long COVID-/Post COVID-Zustand	0,84 (0,75-0,94)		0,93 (0,83-1,04)		0,89 (0,78-1,01)		0,95 (0,85-1,07)		1,00 (0,78-1,27)		0,89 (0,68-1,17)	
	SARS-CoV-2-Infektion, aktuell Long COVID-/Post COVID-Zustand	1,86 (1,61-2,15)		1,36 (1,17-1,59)		1,51 (1,30-1,75)		1,30 (1,12-1,50)		2,40 (1,61-3,58)		1,70 (1,01-2,86)	

Anzahlen ungewichtet, relative Risiken gewichtet.
